# Sex Differences in Associations Between White Matter Microstructure and Cognitive Performance in the Boston Latino Aging Study

**DOI:** 10.1002/alz70856_101384

**Published:** 2025-12-24

**Authors:** Averi Giudicessi, Elouise A. Koops, Nikole A Bonillas Félix, Isabela Gonzalez, Lusiana Martinez, Randy Medrano, Alexandre Closs Fanfa Ribas, Jairo E. Martinez, Jorge Alcina, Isabel Solis, Clara Vila‐Castelar, Marta Gonzalez Catalan, Daniel G Saldana, Liliana A Ramirez Gomez, Yakeel T. Quiroz

**Affiliations:** ^1^ Boston University, Boston, MA, USA; ^2^ Massachusetts General Hospital, Harvard Medical School, Boston, MA, USA; ^3^ Athinoula A. Martinos Center, Massachusetts General Hospital, Boston, MA, USA; ^4^ Massachusetts General Hospital, Department of Radiology, Boston, MA, USA

## Abstract

**Background:**

White matter deterioration is a potential early indicator of neurodegenerative diseases, often preceding cognitive symptoms. These critical brain tracts, essential for inter‐regional communication, are particularly susceptible to early pathological changes. This study examined white matter microstructure and cognitive performance in community‐dwelling older Latinos to understand early neurodegeneration.

**Methods:**

Diffusion‐weighted imaging assessed white matter microstructure in 122 unimpaired Latino older adults from the Boston Latino Aging Study (BLAST). The sample included Latino adults, 55% female, with a mean age of 65 years (SD=7) and average education of 11.8 (SD=4.9). Fractional anisotropy (FA) and mean diffusivity (MD) were extracted with the JHU atlas for 10 major tracts. Global cognitive performance was measured via the Mini‐Mental State Examination (MMSE). Multiple regression analyses examined associations between neuroimaging metrics and cognitive performance with age, sex, and years of education as covariates. Models included an age‐by‐sex interaction term to examine sex‐specific age effects on these relationships.

**Results:**

While accounting for sex differences, significant age related reduced FA was observed in the inferior longitudinal fasciculus (ILF; β=‐0.22, *p* = 0.03) and superior longitudinal fasciculus (SLF; β=‐0.21, *p* = 0.04), as well as increased MD in the anterior thalamic radiation (β=0.35, *p* <0.001), inferior fronto‐occipital fasciculus (β=0.37, *p* <0.001), and cingulum cingulate gyrus (β=0.26, *p* = 0.01). Sex differences were observed in the temporal SLF, with females showing higher FA (β=0.20, *p* = 0.02) and lower MD (β=‐0.21, *p* = 0.03) than males. Global cognitive performance was significantly associated with MD in the hippocampal cingulum (β = ‐0.293, *p* =  0.011), suggesting that higher MMSE scores correlate with greater white matter integrity. No significant associations were found between years of education and diffusion metrics, nor were there any notable sex‐by‐age interaction effects.

**Conclusions:**

This study highlights the vulnerability of white matter to age‐related decline and suggests sex‐specific differences in white matter integrity, especially within language‐related temporal pathways. The association between cognition and hippocampal cingulum integrity emphasizes the potential of diffusion metrics as early biomarkers for cognitive decline. These findings enhance our understanding of the neurobiological mechanisms underlying cognitive aging in Latino populations, and expands our understanding of age related brain integrity changes.

